# Real-World Treatment Landscape of Individuals with Rett Syndrome Treated and Untreated with Trofinetide in the United States

**DOI:** 10.36469/001c.162589

**Published:** 2026-06-11

**Authors:** Nazia Rashid, Vinod K. Yakkala, Safiuddin S. Syed, Krithika Rajagopalan

**Affiliations:** 1 Medical Affairs Acadia Pharmaceuticals, San Diego, California; 2 Anlitiks Inc, Windermere, Florida

**Keywords:** Rett syndrome, trofinetide, treated, untreated

## Abstract

**Background:**

Trofinetide (TROF) became the first approved pharmacologic treatment for Rett syndrome (RTT) in the United States in March 2023; however, real-world evidence comparing TROF-treated and untreated individuals is limited.

**Objective:**

This study aimed to compare the baseline demographic and clinical profiles of those treated with TROF vs untreated in routine clinical practice.

**Methods:**

This retrospective cohort study used linked IQVIA Anonymized Patient Level Database medical claims and a specialty pharmacy database. Individuals with ≥1 medical claim for RTT between 01/01/2021 and 09/30/2024 (study period) were identified. Treated individuals had ≥1 TROF prescription during 04/01/2023 to 09/30/2023 (identification period), with first prescription set as index; untreated individuals had no TROF prescription. A risk-set sampling approach was used to assign proxy index dates to untreated individuals to improve comparability and reduce immortal time bias. Individuals with cerebrovascular disease or brain trauma before RTT diagnosis and those without 12 months pre-and post-index enrollment were excluded. Baseline characteristics were assessed during the 12 months pre-index.

**Results:**

Of 8047 individuals with RTT identified, 2950 met eligibility criteria; 766 (26.0%) were treated with TROF and 2184 (74.0%) remained untreated. Treated individuals were younger at index (15.4 vs 23.5 years), and a greater proportion were pediatric (≤17 years; 66.6% vs 38.3%). Females predominated in both groups, while males represented a smaller proportion of the treated (4.6% vs 10.8%). Treated individuals more frequently had documented nonspecific developmental delay (31.3% vs 21.6%), autism spectrum disorder (19.1% vs 15.7%), and core RTT-related neurodevelopmental features such as loss of acquired communication skills (23.8% vs 12.9%) and loss of acquired motor skills (11.1% vs 4.9%). Child neurology was the most common prescriber specialty in both groups and was more frequent among treated individuals (50.3% vs 26.8%). Overall comorbidity burden was broadly similar between groups.

**Conclusion:**

In this real-world analysis, only one-quarter of eligible individuals with RTT initiated TROF during the early post-approval period. TROF uptake appeared concentrated in younger, specialist-managed individuals with more clearly documented RTT-related features, while three-quarters remained untreated. These findings highlight the need to better understand treatment pathways and barriers to initiation in males and adults in routine RTT care.

## INTRODUCTION

First described by Andreas Rett in 1966 and recognized internationally by Hagberg et al in 1983, Rett syndrome (RTT) is a rare, severe, and progressive X-linked dominant neurodevelopmental disorder commonly caused by pathogenic variants in MECP2 gene.^1,2^ RTT is known to predominantly affect females with an estimated incidence of approximately 1 in 10 000 to 15 000 live female births worldwide.^3–5^ The core diagnostic features of RTT include the loss of purposeful hand skills, loss of spoken language, gait abnormalities, and stereotypic hand movements, and progresses through 4 clinical stages from early developmental stagnation to late motor deterioration.^6–8^ The clinical burden associated with RTT is substantial, often exacerbated by symptoms and other coexisting conditions such as epilepsy, scoliosis, breathing irregularities, constipation, dysphagia, gastroesophageal reflux, and sleep disturbances.^9,10^ Prior to the availability of disease-targeted therapies, management of RTT was confined to symptomatic interventions and supportive care, and both clinicians and caregivers have historically underscored the absence of treatments addressing multiple symptoms simultaneously as a critical unmet need.^11^

Trofinetide (TROF; DAYBUE™), a synthetic analog of glycine-proline-glutamate derived from insulin-like growth factor 1 (IGF-1), was approved by the US FDA in March 2023 as the first and only pharmacological treatment indicated for RTT in patients aged ≥2 years marked an important shift in the RTT treatment landscape.^12,13^ Its approval was based on the results of a phase 3 LAVENDER trial, which demonstrated statistically significant improvements in co-primary endpoints at week 12.^14^ Long-term safety and efficacy were subsequently supported by the open-label LILAC and LILAC-2 extension studies (up to 32 months).^15,16^ Emerging real-world evidence has begun to describe real-world experience with TROF, including early insights into benefits, tolerability, and practical management in routine practice.^17,18^ However, real-world data describing the demographic and clinical profile of individuals with RTT as well as an examination of differences between those who are treated with TROF vs those who remain untreated with TROF are needed to identify gaps in care and inform clinical decision-making. To address this need, the present study utilized linked specialty pharmacy and administrative claims data from a large US database to characterize and compare the baseline demographics and clinical characteristics of individuals with RTT who are either treated or untreated with TROF.

## METHODS

### Study Design, Data Source, and Population

A retrospective cohort study was conducted using linked data comprising the medical claims of individuals with RTT from the IQVIA’s Anonymized Patient Level Database (APLD) and pharmacy data of all patients receiving TROF treatment from a nationally representative specialty pharmacy database. The APLD is an open-source, administrative claims data that includes pre-adjudicated, de-identified healthcare claims data for over 130 million beneficiaries in the United States. The database contains detailed information on demographics, medical claims such as diagnosis, procedures, services, and pharmacy claims, making it a robust real-world data resource to complete the study objectives. On the other hand, the specialty pharmacy data contains dispensing information about TROF treatment among individuals with RTT (eg, date of dispensing, quantity dispensed, days of supply).

The eligible study population derived from the linked database included individuals with at least 1 medical claim for RTT (ICD-10-CM: F84.2) in any diagnostic position from 01/01/2021 to 09/30/2024 (ie, study period). Patients with a diagnosis of cerebrovascular disease (I60.x-I69.x) or brain trauma (S06.x) on or before RTT diagnosis were excluded. The study population was further categorized into 2 groups based on TROF treatment status: those with a TROF prescription (treated group) vs those with no TROF prescription (untreated group).

The index date for the treated group was defined as the first TROF prescription claim observed during the identification period (04/01/2023–09/30/2023). Because untreated individuals did not have a TROF initiation date, a comparable index date was constructed for this group. Assigning an index date to untreated individuals was necessary to align timing between TROF-treated and untreated and to improve comparability between groups. To create an index date for the untreated group, a risk set sampling approach was conducted, which also helped reduce immortal time bias.^19,20^ For each treated individual, the number of days between the first RTT diagnosis and TROF initiation was calculated. A corresponding risk set of untreated individuals was then identified, consisting of patients who had an RTT diagnosis but had not initiated TROF. Up to 5 untreated individuals were randomly selected without replacement for each treated individual and assigned a proxy index date equal to their RTT diagnosis date plus the corresponding treated individual’s time lag from diagnosis-to-treatment interval. This process was repeated iteratively using the remaining untreated individuals until all eligible untreated individuals had been assigned a proxy index date.^19,20^ Additional eligibility criteria included those with at least 12 months of continuous enrollment pre- and post-index periods.

### Study Measures

Baseline demographics and clinical characteristics were assessed during the 12-month pre-index period among both treated and untreated cohorts. Demographic variables evaluated at index date included age, sex (male/female), and geographic region (Midwest, Northeast, South, West). In addition to age at index, age at RTT diagnosis was also assessed. Clinical characteristics included differential diagnosis (eg, autism spectrum disorder, nonspecific developmental delay) and clinical comorbidities (eg, epilepsy, seizures, scoliosis, gastrostomy) and RTT related features (eg, loss of acquired communication skills, loss of motor skills) were also assessed pre-index. Physician specialty (eg, child neurologist, neurologist, pediatrician) was determined based on the claim closest to the index date.

### Statistical Analyses

Categorical variables such as gender, physician specialty, clinical comorbidities were described using frequencies and percentages; continuous variables such as age were described using mean and standard deviation (SD). Between-group comparisons were conducted using 2-sample *t*-tests for continuous variables and chi-square tests using Yates’ continuity correction for categorical variables; Fisher’s exact tests were used when expected cell counts were fewer than 5. No formal imputation of missing data was performed, and statistical significance was defined as a 2-sided *P* value <.05. All analyses were conducted using Anlitiks’ proprietary RapidAnalyzer™ analytic platform, which is powered by SQL and RStudio.

## RESULTS

### Study Population

**Figure 1.**
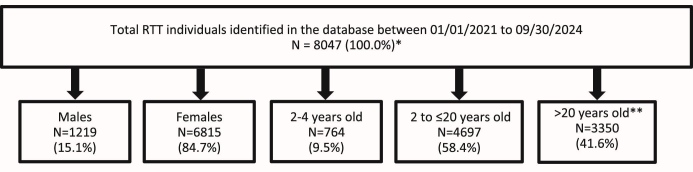
Overall Rett Syndrome Patients and Different Subgroups *In all, 8034 individuals had known gender from the database, 13 were unknown, 1219 were males, and 6815 were females. **The age group >20 years is also known as adults.

**Figure 2.**
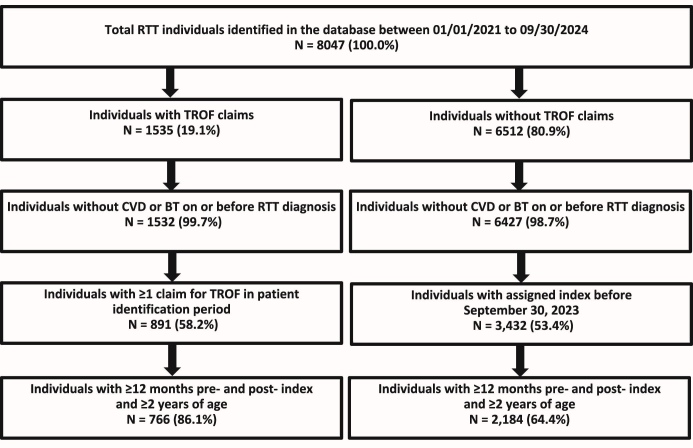
Study Attrition of Individuals with Rett Syndrome Treated and Untreated with Trofinetide Abbreviations: BT, brain trauma; CVD, cerebrovascular disease; RTT, Rett syndrome; TROF, trofinetide. The percentages were calculated based on the previous step; post-index period includes index.

There were 8047 individuals with RTT identified during the study period. The following groups were identified to better understand individuals with RTT: females (84.7%), males (15.1%), 2 to 4 years old (9.5%), 2 to ≤20 years old (58.4%), and adults aged >20 years old (41.6%) (**Figure 1**). The final eligible sample included 2950 individuals (**Figure 2**). Of these, 26.0% (n = 766) were treated with TROF and 74.0% (n = 2184) remained untreated (**Figure 2**).

### Study Measures

Baseline demographic and clinical characteristics are presented in **Table 1**. Treated individuals were substantially younger than untreated individuals at both RTT diagnosis and index. The mean (SD) age at RTT diagnosis was 13.4 (10.3) years in the treated group vs 21.7 (12.8) years in the untreated group; similarly, mean (SD) age at index was 15.4 (10.9) years in the treated group vs 23.5 (12.8) years in the untreated group. Consistent with this pattern, children aged 2 to 17 years accounted for greater proportion of the treated group (66.6% vs 38.3%), while adults aged 18 years or older accounted for majority of untreated individuals (33.4% vs 61.7%), respectively. The majority were female in both groups; however, males comprised a significantly lower proportion of the treated cohort (4.6% vs 10.8%, *P*< .05). Third-party insurance coverage was more common among treated than untreated individuals (67.1% vs 50.5%, *P*<.05), while Medicaid coverage was generally similar between groups (22.3% vs 23.2%, *P* = .67).

**Table 1.** Demographics and Characteristics Among RTT Treated vs Untreated with Trofinetide

	**Overall (N = 2950)**	**Treated (N = 766)**	**Untreated (N = 2184)**	***P***
Age at RTT diagnosis (years)				
Mean, SD	19.7, 12.8	13.4, 10.3	21.7, 12.8	<.05
Median, IQR	17, 18	11, 12	19, 18	
Age at TROF index (years)				
Mean, SD	21.4, 12.9	15.4, 10.9	23.5, 12.8	<.05
Median, IQR	19, 18	13, 13	21, 18	
Age categories at TROF, n (%)				
Pediatric				
2-4 years	129 (4.4)	94 (12.3)	35 (1.6)	<.05
5-10 years	529 (17.9)	208 (27.2)	321 (14.7)	<.05
11-17 years	689 (23.4)	208 (27.2)	481 (22.0)	<.05
Adult				
18-20 years	293 (9.9)	78 (10.2)	215 (9.8)	.84
21-29 years	583 (19.8)	106 (13.8)	477 (21.8)	<.05
30-39 years	424 (14.4)	41 (5.4)	383 (17.5)	<.05
40-49 years	214 (7.3)	18 (2.4)	196 (9.0)	<.05
≥50 years	89 (3.0)	13 (1.7)	76 (3.5)	<.05
Gender, n (%)				
Female	2680 (90.8)	731 (95.4)	1949 (89.2)	<.05
Male	270 (9.2)	35 (4.6)	235 (10.8)	
Region, n (%)				
South	965 (32.7)	263 (34.3)	702 (32.1)	.29
Midwest	651 (22.1)	223 (29.1)	428 (19.6)	<.05
West	572 (19.4)	140 (18.3)	432 (19.8)	.39
Northeast	253 (8.6)	72 (9.4)	181 (8.3)	.38
Unknown/unspecified	509 (17.3)	68 (8.9)	441 (20.2)	<.05
Differential diagnosis, n (%)				
Nonspecific developmental delay	711 (24.1)	240 (31.3)	471 (21.6)	<.05
Autism spectrum disorder	488 (16.5)	146 (19.1)	342 (15.7)	<.05
Cerebral palsy	622 (21.1)	117 (15.3)	505 (23.1)	<.05
Insurance type, n (%)				
Third-party	1,616 (54.8)	514 (67.1)	1,102 (50.5)	<.05
Medicaid	677 (22.9)	171 (22.3)	506 (23.2)	.67
Medicare Part D	359 (12.2)	51 (6.7)	308 (14.1)	<.05
Cash	98 (3.3)	17 (2.2)	81 (3.7)	.06
Unknown	192 (6.5)	13 (1.7)	179 (8.2)	<.05

Abbreviations: IQR, interquartile range; NA, not applicable; RTT, Rett syndrome; SD, standard deviation.

**Figure 3.**
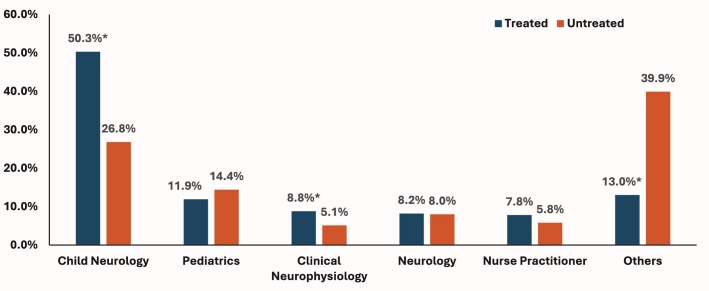
Prescriber Specialty for Treated vs Untreated Individuals with Rett Syndrome A total of 1895 individuals with RTT had a specialty identified closest to their index date: treated = 766 individuals with RTT (100%); untreated = 1129 individuals with RTT (51.7%). Others included family medicine, internal medicine, physician assistant, etc. *P < .05.

Differential diagnoses were significantly more frequent among treated vs untreated individuals: nonspecific developmental delay (31.3% vs 21.6%, *P*< .05) and autism spectrum disorder (19.1% vs 15.7%, *P*< .05). In contrast, cerebral palsy was less prevalent in the treated group (15.3% vs 23.1%, *P*< .05). Among individuals with known prescriber specialty, child neurology was the most common specialty in both groups but was more frequent among treated than untreated individuals (50.3% vs 26.8%, *P*< .05). Clinical neurophysiology was also more common among treated individuals (8.7% vs 5.1%, *P*< .05), whereas pediatrics, neurology, and nurse practitioner representation were similar between groups (**Figure 3**).

The comorbidity profile, summarized in **Table 2**, was broadly similar between groups. Common gastrointestinal conditions in treated vs untreated cohorts included gastrostomy (28.7% vs 29.3%, *P*= .78), dysphagia (28.1% vs 25.4%, *P*= .15), and constipation (25.5% vs 25.0%, *P*= .80), with no statistically significant between-group differences. Musculoskeletal and respiratory comorbidities including scoliosis (24.5% vs 24.5%, *P*= 1.00), respiratory failure (14.0% vs 15.6%, *P*= .28), and cough (13.7% vs 13.5%, *P*= .86) were comparable. History of epilepsy was the most prevalent overall and within both cohorts (46.0% vs 50.9%, *P*< .05), whereas rates of seizures were similar between treated vs untreated (24.3% vs 23.7%, *P*= .75). Infectious conditions such as upper respiratory infection (17.5% vs 13.8%, *P* < .05) and influenza (5.2% vs 2.6%, *P*< .05) were more common in the treated group.

**Table 2.** Comorbidities Among Individuals with Rett Syndrome Treated vs Untreated with Trofinetide

	**Overall (N = 2950)**	**Treated (N = 766)**	**Untreated (N = 2184)**	***P*** ^a^
Gastrointestinal disorders, n (%)				
Gastrostomy	859 (29.1)	220 (28.7)	639 (29.3)	.78
Dysphagia	770 (26.1)	215 (28.1)	555 (25.4)	.15
Constipation	741 (25.1)	195 (25.5)	546 (25.0)	.80
Gastroesophageal reflux disorder	485 (16.4)	135 (17.6)	350 (16.0)	.30
Vomiting	216 (7.3)	55 (7.2)	161 (7.4)	.86
Infectious/viruses, n (%)				
Upper respiratory infection	436 (14.8)	134 (17.5)	302 (13.8)	<.05
Lower respiratory tract infection	360 (12.2)	96 (12.5)	264 (12.1)	.75
Fever	336 (11.4)	94 (12.3)	242 (11.1)	.37
COVID-19	173 (5.9)	54 (7.0)	119 (5.4)	.10
Urinary tract infection	216 (7.3)	48 (6.3)	168 (7.7)	.19
Influenza	97 (3.3)	40 (5.2)	57 (2.6)	<.05
Musculoskeletal disorders, n (%)				
Scoliosis	724 (24.5)	188 (24.5)	536 (24.5)	1.00
Neurological disorders, n (%)				
Epilepsy	1464 (49.6)	352 (46.0)	1112 (50.9)	<.05
Seizures	704 (23.9)	186 (24.3)	518 (23.7)	.75
Respiratory disorders, n (%)				
Respiratory failure	448 (15.2)	107 (14.0)	341 (15.6)	.28
Cough	399 (13.5)	105 (13.7)	294 (13.5)	.86
Aspiration	274 (9.3)	82 (10.7)	192 (8.8)	.12
Asthma	279 (9.5)	70 (9.1)	209 (9.6)	.73

A total of 1895 individuals with RTT had a specialty identified closest to their index date: treated = 766 individuals with RTT (100%) and untreated = 1129 individuals with RTT (51.7%). Others included family medicine, internal medicine, physician assistant etc.

^a^*P*< .05 was defined as statistically significant.

RTT-related features, presented in **Table 3**, show that neurodevelopmental features were more frequently documented in treated individuals than untreated individuals: loss of acquired communication skills (23.8% vs 12.9%, *P*< .05), loss of acquired motor skills (11.1% vs 4.9%, *P*< .05), delayed developmental progress (8.4% vs 5.0%, *P*< .05), and prominent hand apraxia/dyspraxia (8.1% vs 3.8%, *P*< .05). In contrast, nutritional deficiency (17.2% vs 16.9%, *P*= .85) and breathing irregularities (13.8% vs 13.6%, *P*= .87) were similar among treated vs untreated individuals.

**Table 3.** RETT Related Features Among Individuals with RTT, Treated vs Untreated with Trofinetide

	**Overall (N = 2950)**	**Treated (N = 766)**	**Untreated (N = 2184)**	***P***
Growth abnormalities/nutritional disorders, n (%)				
Nutritional deficiency	502 (17.0)	132 (17.2)	370 (16.9)	.85
Neurodevelopmental disorders, n (%)				
Loss of acquired communication skills	463 (15.7)	182 (23.8)	281 (12.9)	<.05
Weakness/paralysis	450 (15.3)	131 (17.1)	319 (14.6)	.10
Behavioral disorders/symptoms^a^	331 (11.2)	89 (11.6)	242 (11.1)	.68
Loss of acquired motor skills	192 (6.5)	85 (11.1)	107 (4.9)	<.05
Development progress delayed	173 (5.9)	64 (8.4)	109 (5.0)	<.05
Sleep dysfunction	255 (8.6)	63 (8.2)	192 (8.8)	.63
Prominent hand apraxia/dyspraxia	146 (4.9)	62 (8.1)	84 (3.8)	<.05
Respiratory disturbances, n (%)				
Breathing irregularities	403 (13.7)	106 (13.8)	297 (13.6)	.87

^a^Including irritability, aggressive behavior, crying tantrums, self-mutilation, scratching, biting.

## DISCUSSION

This retrospective real-world data analysis is the first study to characterize the demographic and clinical profile of all individuals with RTT who initiated TROF compared with those who remained untreated with TROF during the initial post-approval study period in the United States. Upon FDA approval of TROF, we found approximately one-quarter of individuals with RTT initiated TROF during the identification period, while the remaining three-quarters remained untreated. This finding is notable in the context of RTT’s substantial multisystem burden and the historically limited treatment landscape, in which management has relied largely on symptomatic and supportive interventions.^10,11^ Although TROF changed the treatment landscape as the first approved pharmacologic therapy for RTT, the uptake in real-world practice appears to have been slow during TROF’s early post-approval period.^14^

In this analysis, TROF treated individuals were younger in age than untreated individuals. Approximately three-quarters of TROF treated individuals were children aged <18 years, whereas nearly three-quarters of untreated individuals were adults (≥18 years) at index date. This pattern is directionally consistent with the clinical development program for TROF, in which the pivotal LAVENDER trial enrolled females aged 5 to 20 years, and with subsequent extension studies that likewise largely reflected pediatric and adolescent populations.^14–16,21^ The younger age profile among treated individuals may therefore reflect early adoption in populations and care settings most aligned with the initial trial experience, rather than uniform uptake across the full age spectrum of RTT. This interpretation is also consistent with recent real-world RTT data showing that supportive and pharmacologic interventions are often more intensively used in pediatric than adult populations.^11^

The strong predominance of females in both cohorts was expected, but the relatively larger proportion of males in the untreated group suggests that treatment uptake may be less established outside the classic female RTT population. Because most controlled TROF data were generated in females, clinicians may have initially been more comfortable prescribing within that evidence base.^14^ Accordingly, the lower real-world adoption among males may be conservative due to many factors such as unfamiliarity with treatment protocols for males, residual diagnostic complexity, or both.

This study also suggests that treated and untreated individuals differ not only by age but also by the different diagnosis journey among individuals with RTT. Treated individuals more frequently had history of nonspecific developmental delays, autism spectrum disorder, and several core RTT-related neurodevelopmental features documented, including loss of acquired communication and motor skills, delayed developmental progress, and hand apraxia/dyspraxia. These findings may indicate that individuals with more clearly documented RTT-related phenotypes were more likely to receive TROF in routine care. The higher representation of child neurology and clinical neurophysiology among treated individuals further supports the possibility that specialist involvement may play an important role in both recognition of RTT-associated features and treatment initiation. Given that diagnosis is often made by neurologists and pediatricians rather than primary care providers, differences in specialist involvement may plausibly influence both treatment initiation and documentation patterns.^22^ The TROF clinical program also enrolled individuals with RTT from Centers of Excellence sites, and this could also play a role in knowledge of TROF treatment and care for RTT. Therefore, it is possible that differences between treated and untreated individuals in reported rates of RTT-related neurodevelopmental features and comorbidities may reflect differential diagnostic intensity and specialist-level coding practices rather than clinical severity differences. Therefore, these descriptive results should be interpreted with caution regarding the clinical heterogeneity between groups.

In contrast, overall comorbidity burden was broadly similar between treated and untreated cohorts. Common gastrointestinal, respiratory, musculoskeletal, and neurologic comorbidities were prevalent in both groups, underscoring the high baseline burden of RTT regardless of treatment status. This is consistent with prior natural history and burden-of-illness literature showing that constipation, feeding and swallowing difficulties, epilepsy, scoliosis, respiratory dysfunction, and sleep-related problems remain common across the lifespan in RTT.^10,11^ This suggests that, during the early post-approval period, TROF use may have been differentiated more by age, care pathway, and recognition of RTT-related clinical features than by comorbidity burden alone.

The findings of this study should be interpreted in the context of limitations inherent to real-world claims analyses using claims data, which may be subject to miscoding errors. Consistent with prior published real-world RTT research, this analysis also identified RTT diagnosis as those with at least 1 diagnostic claim for ICD-10-CM code F84.2 in any diagnostic position.^23^ While diagnostic misclassification is less likely in those who initiated TROF, it is possible that the untreated population may be subjected to miscoding errors. To address this limitation, however, the untreated population was matched with treated patients using a risk-stratified sampling to ensure similar person-time exposure and were comparable.

Since age at RTT diagnosis was based on the first RTT diagnostic claim in the database, it is possible that true age at RTT clinical diagnosis may be different for some individuals. Although risk-stratified sampling resulted in comparable groups, confounding due to unobservable variables in the claims data that may influence clinical decision-making (ie, genetic testing, functional severity, caregiver preferences, or quality-of-life data) may not be ruled out. To the extent that at least 12 months of continuous pre- and post-index enrollment excluded individuals lacking stable insurance coverage and care continuity, it may underrepresent individuals with interrupted coverage, shorter observable follow-up, or less consistent healthcare utilization. Notwithstanding these limitations and the need to exercise caution in interpreting these descriptive results as indicators of group differences rather than evidence of causal associations, these group differences represent an important finding that may require further investigation in future. Physician specialty was assigned based on the claim closest to the index date and may not fully reflect longitudinal multidisciplinary care. Nonetheless, the use of linked specialty pharmacy and administrative claims data is a key strength, as it enabled characterization of treatment exposure alongside real-world demographic and clinical profiles in a large US RTT population during the initial phase of TROF uptake.

Overall, these descriptive findings suggest that TROF adoption and utilization patterns in the post-FDA approval period was concentrated in younger, specialist-managed, predominantly female patients and did not include the broad population of RTT. These results have broad implications for clinical practice patterns and treatment decision-making. First, there is a need to improve awareness and treatment consideration among adult care providers, as many untreated individuals were adults who may fall outside traditional pediatric RTT care pathways. Second, the lower uptake observed among males highlights the importance of ensuring that treatment consideration is guided by clinical need and current labeling rather than historical patterns of recognition or trial representation. Third, the large untreated population indicates an opportunity to better understand and address practical barriers to uptake, including referral patterns, prescriber familiarity, diagnostic recognition, and potential access-related challenges. Future studies should further evaluate treatment pathways, provider specialty patterns and barriers to initiation in both adults and males with RTT, while also assessing long-term outcomes and healthcare resource utilization associated with TROF in routine care.

## CONCLUSION

In this real-world claim analysis of individuals with Rett syndrome, only 26% of eligible individuals initiated TROF while 74% remained untreated, underscoring a substantial unmet treatment need across the RTT population. Baseline comorbidity burden was broadly comparable between treated and untreated individuals, with high rates of epilepsy, gastrointestinal disorders, and musculoskeletal conditions in both groups suggesting that clinical complexity alone does not fully explain treatment initiation decisions. The treated group had significantly higher RTT-defining clinical features, including loss of acquired communication and motor skills, developmental delay, and hand apraxia/dyspraxia, potentially reflecting greater clinical severity or more thorough specialty-level assessment driving TROF initiation. Treated individuals were also more often managed by neurologic specialists, underscoring the potential importance of specialty involvement in facilitating TROF initiation. Overall, these findings suggest that treatment uptake in RTT may be influenced more by age, phenotypic recognition, and care pathway than by comorbidity burden alone. Future studies should further evaluate healthcare resource utilization and barriers to treatment initiation to better understand the real-world impact of TROF in RTT.

### Disclosures

Nazia Rashid is an employee of Acadia Pharmaceuticals. Vinod Kumar Yakkala, Safiuddin Shoeb Syed, and Krithika Rajagopalan are employees of Anlitiks Inc., which received funding from Acadia Pharmaceuticals to conduct this study.
